# Frontiers in antimicrobial stewardship: antimicrobial use during end-of-life care

**DOI:** 10.1017/ash.2023.207

**Published:** 2023-10-02

**Authors:** Seohyuk Lee, Rupak Datta

**Affiliations:** 1 Department of Internal Medicine, Yale School of Medicine, New Haven, CT, USA; 2 Veterans Affairs Connecticut Healthcare System, West Haven, CT, USA

Improving the use of antimicrobials across healthcare settings is a national priority. While considerable literature has accumulated regarding antimicrobial stewardship across the continuum of care, new frontiers for implementation remain.^1^ Antimicrobial stewardship during end-of-life care is unique because its principles are employed in the context of palliative care. Understanding how antimicrobials facilitate palliative care – with its focus on management of symptoms, psychosocial support, and assistance with decision-making – offers new opportunities to optimize the reach and effectiveness of antimicrobial stewardship.^2^ Nevertheless, many aspects of this area warrant increased scrutiny by stakeholders.

## End-of-life care

There is no standardized definition of end-of-life.^3^ In general, end-of-life refers to the final days to weeks of a life-limiting illness. However, alternative definitions encompass the entire time interval of a life-limiting illness, such as advanced cancer or advanced dementia, when death would not be unexpected.^4^ Palliative care can complement curative therapies during the end-of-life period and may be delivered by diverse clinicians (eg, physicians and advanced practice providers) and healthcare settings (eg, acute care, long-term care, and home-based care).^5^ Such heterogeneity in the provision of end-of-life care can complicate the implementation of effective antimicrobial stewardship.

## Estimates of antimicrobial use

Patients near the end-of-life are prone to infection due to the prevalence of immunosuppression, multimorbidity, cognitive impairment, and device utilization.^6–9^ Consequently, exposure to antimicrobials is common during palliative care. Among hospitalized patients experiencing cancer-related death, 87% received antimicrobials during hospitalization, and over one-third of these patients received antimicrobial therapy following transition to comfort care.^10,11^ Among nursing home residents with advanced dementia, more than 40% received antimicrobials in the 2 weeks prior to death.^12^ Nationally, 27% of hospice patients received ≥ 1 antimicrobial during the last week of life, and over 1 in 5 patients discharged to hospice were continued on antimicrobials.^13,14^ In one recent meta-analysis, based on data from 72 studies in which the definition of end-of-life ranged from the day of death to 6 months prior to death, over 50% of patients near the end-of-life receive antimicrobials across healthcare settings.^15^ Importantly, evidence to support the presence of bacterial infection was insufficient in most studies, suggesting that many antimicrobial prescriptions are potentially inappropriate.^16,17^ These data indicate that exposure to antimicrobial therapy is substantial during end-of-life care and establish ripe targets for future research and quality improvement.

## Aligning antimicrobial therapy with goals of care

Goals of care often vary from survival to comfort near the end-of-life. Yet, to date, no study has rigorously evaluated the impact of antimicrobial therapy on mortality or relief of symptoms in an end-of-life population. Two systematic reviews provided limited data to support the use of antimicrobial therapy to achieve relief of symptoms among patients receiving palliative care.^15,18^ It remains unknown what specific symptoms associated with infection are most likely to benefit from antimicrobial therapy during this period. Limited evidence suggests that genitourinary symptoms related to urinary tract infection may improve with antimicrobial therapy, whereas those associated with oral cavity, skin and soft tissue, and bloodstream infections may be less responsive.^19,20^ With respect to respiratory symptoms, there are conflicting data. In one American study of patients with advanced dementia and suspected pneumonia, antimicrobial therapy was associated with decreased comfort but improved survival.^21^ In contrast, in two Dutch studies, antimicrobial therapy was associated with lower symptomatic burden among patients with dementia who developed pneumonia.^22,23^ These data suggest that the use of antimicrobial therapy for the symptomatic management of infection may lack benefit in long-term care and hospice settings. In acute care settings, withholding antimicrobials should be considered when survival is not a primary goal given the high potential for harm and limited data on efficacy related to relief of symptoms.^24^ Ultimately, antimicrobial therapy should be deemed aggressive care during the end-of-life period and be administered orally whenever possible based on good practice recommendations.^24^

## Behavioral and decision-making aspects

It is likely that behavioral and decision-making aspects are key barriers to the implementation of antimicrobial stewardship during end-of-life care. Despite the substantial harms associated with antimicrobial therapy, such as adverse drug events, *Clostridioides difficile* infection, and antimicrobial resistance, the pressures to prescribe are powerful and often multifactorial. For example, among 283 surveyed physicians affiliated with an academic medical center, 86% and 75% continued antimicrobial therapy during end-of-life care to honor the request of patients and family members, respectively.^25^ These providers often cited a desire to avoid the perception that they were giving up on the patient.^25^ Among patients discharged to hospice, nearly 20% of prescriptions were linked to the specific desire of patients and/or their family members to receive antimicrobial treatment.^26^ Additionally, the decision to withhold or withdraw antimicrobial therapy may be sensitive to social dynamics within interdisciplinary care teams, including hierarchy, professional power, and shared accountability.^27–29^ These factors, along with many others (eg, fear of negative patient satisfaction scores, perceived burden of treatment, institutional culture, and ethical aspects of end-of-life care) lie in the backdrop of prognostic uncertainty.^25,30,31^ Given that predicting death is inevitably imprecise, physicians may favor continuing antimicrobials among patients receiving end-of-life care.

## Future directions

There are many potential pathways to promote antimicrobial stewardship during end-of-life care. Good practice recommendations emphasize shared decision-making about future care and agreement regarding goals of treatment as part of advance care planning.^32^ These recommendations, combined with recent survey data, underscore a role for educational programs (eg, training modules and communication simulation exercises) to increase the integration of antimicrobial use into advance care planning at the time of enrollment in long-term care or hospice programs.^33,34^ At the facility level, multifaceted interventions supported by information technology including antimicrobial restrictions, clinical decision support tools, and/or comfort care order sets may be designed and evaluated specifically for patients receiving end-of-life care in acute care settings. The benefits and harms of antimicrobial use during end-of-life care using valid and reliable metrics involving patient and caregiver relevant outcomes also require investigation across racially and ethnically diverse populations. Additionally, there is a need to integrate best practices related to antimicrobial stewardship, such as the “Four Moments of Antibiotic Decision Making,” into palliative care settings; this may be achieved using methods of implementation science.^35,36^ Finally, there are no national or international guidelines to facilitate decision-making related to antimicrobial use during end-of-life care. Future studies may consider addressing this gap in knowledge using methods that combine expert opinion and evidence in a systematic manner.^37^

In conclusion, antimicrobial use is prevalent during end-of-life care. As antimicrobial stewardship programs strive to optimize antimicrobial prescribing across the continuum of care, end-of-life care represents a challenging new frontier for antimicrobial stewards to improve clinical outcomes and reduce antimicrobial-associated harms.
